# Moisture Sorption Isotherms of Sweet Cherry (*Prunus Avium* L.): Comparative Study of Kinetics and Thermodynamic Modeling of Five Varieties

**DOI:** 10.1155/2022/6786590

**Published:** 2022-10-26

**Authors:** Rachida Ouaabou, Said Ennahli, Lahcen Hssaini, Bouchra Nabil, Ali Idlimam, Abdelkader Lamharrar, Mostafa Mahrouz, Hafida Hanine

**Affiliations:** ^1^Department of Chemistry, Faculty of Sciences Semlalia, Cadi Ayyad University, 40000 Marrakech, Morocco; ^2^Faculty of Sciences and Technology, Sultan Moulay Slimane University, BO 523, Beni-Mellal, Morocco; ^3^National School of Agriculture of Meknes, ENAM, Morocco; ^4^National Institute of Agricultural Research, Avenue Ennasr, BP 415 Rabat Principale, 10090 Rabat, Morocco; ^5^High School of Technology, University Sultan Moulay Slimane, Beni-Mellal, Morocco; ^6^Team of Solar Energy and Aromatic and Medicinal Plants, EESPAM, ENS, Laboratory of Processes for Energy, & Environment ProcEDE, Cadi Ayyad University Marrakesh, Morocco

## Abstract

Moisture sorption isotherms of five sweet cherry cultivars (*Prunus avium* L.) at three temperatures of 30°C, 40°C, and 50°C, and water activity range of 0.057–0.898 were determined using the static gravimetric method. The sorption isotherms of all cultivars decreased with increasing temperature, and they all exhibited type II behavior according to the classification of IUPAC (International Union of Pure and Applied Chemistry). The isosteric heat of sorption, differential entropy, spreading pressure, and water surface area were determined, and the energy associated with the sorption processes was defined. The curves were fitted to GAB, PELEG, and ENDERBY models, and the GAB model gave the best fit for the whole set of data. The enthalpy–entropy compensation proved that the process occurs spontaneously and is fully controlled the enthalpy. The spreading pressure value varied with temperature in all sweet cherry cultivars in both the desorption and adsorption processes. The average surface area varied from 78.05 to 214.02 m^2^/g for desorption and from 49.0 to 204.4 m^2^/g for adsorption from 30 to 50°C.

## 1. Introduction

Sweet cherry is a nonclimacteric and highly perishable fruit known for its high water content in tissues, thin skin, and high respiration rate [1]. Thanks to their excellent taste, high nutritional value, and lower caloric content, these cherries are highly prized by consumers. The fruit contains significant levels of nutrients such as dietary fiber, ascorbic acid, carotenoids, anthocyanins, and phenolic acids [2, 3]. Additionally, sweet cherries are reported to contain phenolic compounds and anthocyanins [4, 5]. The health benefits of cherries are well established. For example, cherry consumption reduces the risk of numerous degenerative diseases, such as cancer and cardiovascular diseases [4]. Consumption can also cause a significant reduction of arthritis and gout pain [6]. Fresh sweet cherries are highly perishable and very prone to microbial spoilage, even at low temperatures. Drying is a more practical and low-cost technique to preserve sweet cherries and it is one of the best practices for preservation and extending fruit shelf life [7]. Properly dried fruits are dehydrated to a moisture content that makes the fruit safe to store for prolonged periods of time. In remote rural areas of North African countries, open-air sun drying is most common method for drying sweet cherries. However, a major drawback of this technique is that high volumes of product are lost to inadequate drying, fungal growth, and the encroachment of insects, birds, rodents, etc. Moreover, the final product is usually of poor quality [8, 9] and unhygienic as a result of microorganisms and insects present [10].

Due to the recent increase in demand for and large-scale production of sweet cherries and the need for an efficient, low-cost, and effective drying technique, it is critical that the open-air sun drying is replaced with a more efficient drying system. In order to best control the drying process and storage conditions, it is necessary to know the relationship between the equilibrium moisture content (EMC) in sweet cherries and the equilibrium relative humidity (ERH) of the drying air at a given temperature. This relationship is typically described by the moisture sorption isotherm equations. The moisture sorption isotherm of food graphically relates its equilibrium moisture content in either desorption or adsorption to the water activity (*a_w_*) at a definite temperature [11].

The sorption isotherm properties of food and agricultural products are therefore of special interest in the food industry especially for designing storage and preservation processes such as packaging, storing, mixing, drying, freeze-drying, as well other processes that involve the prediction of food stability and shelf life, texture, and alterations kinetics in food and agricultural [12–15]. Furthermore, sorption isotherms can be used to calculate the enthalpy, entropy, and free-energy values needed for food preservation [16].

This study determined the desorption and adsorption moisture isotherms of five sweet cherry fruit cultivars at drying temperatures of 30, 40, and 50°C. Then, it evaluated the suitability of commonly used moisture isotherm model equations. The nature of the moisture sorption hysteresis was reported, as well as the determination of thermodynamic properties (differential enthalpy, differential entropy, Gibbs free energy, spreading pressure, and surface area).

## 2. Materials and Methods

### 2.1. Raw Material

The fruits from the five most cultivated sweet cherry cultivars: Burlat, Van, Cerisette, Napoleon, and Cœur pigeon in the regions of Marrakech and Meknes were harvested at their full maturity. Fruits of uniform size and color were selected. The fruits were kept in polyethylene bags and transported in portable cooler. The samples mass varied 5.24 g and 8.72 g. The samples moisture was measured using the AOAC (1995) vacuum oven method [17]. Then, the soluble solids content (SSC) was determined with a refractometer (DR6000, Hamburg, Germany) and expressed as °Brix.

### 2.2. Sorption Isotherms Experiments

**Figure 1:**
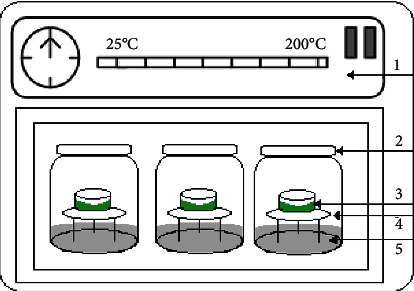
Experimental apparatus for the sorption isotherms measurement: (1) thermostated bath; (2) glass jar containing salt solution; (3) sample holder; (4) sample; (5) saturated salt solution.

Moisture sorption isotherms were derived using the static gravimetric method (Figure 1), the adsorption and desorption equilibrium moisture contents of the five sweet cherry cultivars were determined at temperatures of 30, 40, and 50°C, which are mostly used for agricultural products. The salts water activity (*a_w_*) ranged from 0.074 to 0.898, 0.063 to 0.891, and 0.057 to 0.882 at 30, 40, and 50°C, respectively (Table 1). This method relies on the use of saturated salt solutions to maintain a constant relative humidity level in enclosed, still, and moist air at a specific temperature to generate the complete sorption isotherms. For adsorption isotherms, fruit samples were air-dried at 50°C to a water activity less than 0.08. For desorption isotherms, fresh cherries were used. A sample quantity of 2 g (desorption) 0.5 g (adsorption) were placed on glass tripods inside 1 L sealed glass desiccator jars containing saturated salt solutions (Figure 1). These jars were placed inside temperature-controlled ovens set at 30, 40, and 50°C. The samples were weighed daily until three identical consecutive measurements were observed which is equivalent to the final equilibrium. About 8 to 10 days were required for equilibration of desorption and adsorption, respectively. The dry matter content (*M_s_*) was measured by oven drying the sample at 105°C for 24 hours [18, 19]. Each experiment was carried out in triplicate.

The equilibrium moisture content EMC of the sample is calculated by applying the following:

(1) $$\begin{array}{c} \text{EMC}=X_{\text{eq}}=\frac{M_{h}-M_{s}}{M_{s}}, \end{array}$$

where *M*_*h*_ is the mass of the sample before drying, *M*_*s*_ is the mass of the sample after drying, and *X*_*e*_ is the equilibrium moisture content.

### 2.3. Modeling Equations

The adsorption and desorption equilibrium moisture contents of the five cherry cultivars were fitted to three of the moisture sorption isotherm models presented in Table 2. The GAB, PELEG, and ENDERBY models were fitted using nonlinear regression to minimize the sum of squares of deviations between experiment and theory in a series of iterative steps. All calculations were made using Curve Expert Professional. The suitability of the suitable model is based on the minimum values of the mean relative error (MRE) and the maximum of the correlation coefficient (*r*). Using the correlation coefficient (*r*), the best fit model was tested using the mean relative error (MRE): These parameters are defined as follows:

(2) $$\begin{array}{c} r=\sqrt{\frac{\sum_{i=1}^{N}\left(X_{{\text{eq}}_{\text{i},\text{pred}}}-{\overset{¯}{X}}_{{\text{eq}}_{\text{i},\exp}}\right)2}{\sum_{i=1}^{N}\left(X_{{\text{eq}}_{\text{i},\exp}}-{\overset{¯}{X}}_{{\text{eq}}_{\text{i},\exp}}\right)2}},\\ \text{MRE}=\frac{100}{N}=\overset{N}{\underset{i=1}{\sum }}\left|\frac{X_{{\text{eq}}_{\text{i},\exp}}-X_{{\text{eq}}_{\text{i},\text{pred}}}}{X_{{\text{eq}}_{\text{i},\exp}}}\right|, \end{array}$$

where *X*_eq_i, exp is the experimental moisture content (% d.b), *X*_eq_i, pred is the predicted moisture content (% d.b), *N* number of data points, and *d*.*b* is the dry weight basis. The model is considered satisfactory if the average of the *N* values is less than or close to 10% [20].

### 2.4. Thermodynamic Properties

At constant moisture content, the net isosteric heat, *q*_st_ (kJ mol^−1^), was calculated using the equation derived from the Clausius–Clapeyron equation:

(3) $$\begin{array}{c} q_{\text{st}}=-R\left[\frac{d\left(\ln{\text{a}}_{w}\right)}{d\left(1/\theta \right)}\right]X_{\text{eq},} \end{array}$$

where *a*_*w*_ is the water activity (dimensionless), *θ* is the absolute temperature (K), and *R* is the universal gas constant (J/(mol K)).

By assuming that the net isosteric heat is invariable with the temperature for a given EMC, the integration of the Equation (3) gives [21]

(4) $$\begin{array}{c} \ln{\text{a}}_{w}=\frac{-q_{\text{st}}}{R}\frac{1}{T}+\frac{∆S}{R}, \end{array}$$

where *ΔS* is the differential entropy of sorption (J/kmol).

The plot of lna*_w_* versus 1/*T* gives a straight line with slope of *q*_st_/*R* and intercept of *ΔS/R*. Thus, the isosteric heat of sorption *q*_st_ and the differential entropy *ΔS* could be evaluated from the respective slope and intercept.

The Gibbs free energy is calculated from the equation of Gibbs-Helmholtz as follows [22]:

(5) $$\begin{array}{c} \Delta G=\text{RTLn}\left(a_{w}\right). \end{array}$$

According to the compensation theory [23], the linear relation between enthalpy and entropy for a specific reaction is given by the following equation:

(6) $$\begin{array}{c} q_{\text{st}}=T_{\beta }\Delta S+{\Delta G}_{\beta }. \end{array}$$

The isokinetic temperature *T*_*β*_, represents the temperature at which all reactions in the series proceed to the same rate. The sign of free energy (Δ*G*_*β*_) values at *T*_*β*_ can be used as an indicator of the spontaneity of sorption process. It is considered spontaneous if Δ*G*_*β*_ is negative (−Δ*G*_*β*_) and nonspontaneous if it is positive (+Δ*G*_*β*_).

The thermodynamic properties are evaluated at the constant spreading pressure, which equals the force applied in the plane of the surface to keep the surface from diffusion [24]. It is the surface excess energy and is considered as the increase in surface tension of bare sorption sites due to sorbed molecules [25]. This key parameter is estimated via an analytical procedure involving both moisture content and water activity.

(7) $$\begin{array}{c} \pi =\frac{{\text{K}}_{B}T}{A_{m}}\int_{0}^{a_{w}}\frac{\theta }{a_{w}}{\text{da}}_{w}, \end{array}$$

where *k*_*B*_ is Boltzmann constant (1.380 × 10^−23^ JK^−1^), *A*_*m*_ is the area of a water molecule (1.06 × 10^−19^ m^2^), and the moisture ratio *θ* is given by *θ* = *X*_eq_/*X*_*m*_, *T* (*K*) temperature.

Based on GAB model formula and substituting of *θ*/*a_w_*, the integral incorporated spreading pressure can be determined using GAB model, the substitution and integration in (Equation (7)) can be tackled analytically resulting in the mathematical definition for *π*

(8) $$\begin{array}{c} \pi =\frac{K_{B}T}{A_{m}}\ln\left[\frac{1-B_{a_{w}}+C_{a_{w}}}{1-C_{a_{w}}}\right], \end{array}$$

where *B* and *C* are the constants of GAB model.

The values of water surface area, given in m^2^ g^−1^ of solid, can be determined from Equation (9), using the monolayer moisture values [26].

(9) $$\begin{array}{c} S_{0}=M_{m}\times \frac{1}{{\text{PM}}_{H2O}}\times N_{0}\times A_{H2O}=3.5\times 10^{3}\times M_{m}, \end{array}$$

where *S*_0_ is the surface area (m^2^ g^−1^), *M*_*m*_ is the monolayer moisture content, PM_*H*2*O*_ is the molecular weight of water (18 g mol^−1^), *N*_0_ is the number of Avogadro (6 × 1023 molecules per mole), and *A*_*H*2*O*_ is the area of water molecule (10.6 × 10^−20^ m^2^).

## 3. Results and Discussion

### 3.1. Desorption and Adsorption Isotherms

**Figure 2:**
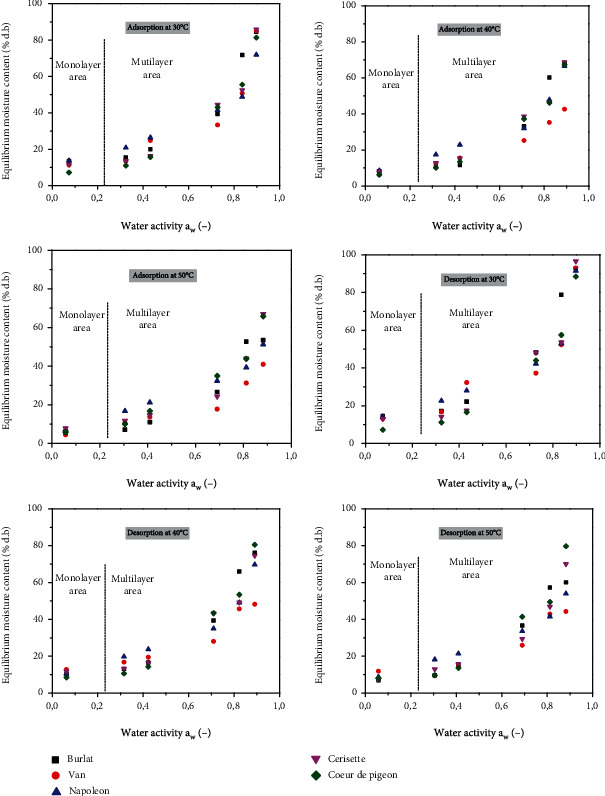
Desorption and adsorption isotherms of cherry cultivars at 30°C, 40°C, and 50°C.

The experimental data of equilibrium water content versus water activity performed on five sweet cherry cultivars at 30, 40, and 50°C are presented in Table 3 and Figure 2.

The desorption-adsorption isotherms given in Figure 2 present the sigmoidal-shaped profile according to IUPAC classification. These curves are typical of most fruits with high amounts of sugar, polysaccharides, and proteins [27]. Sugar content, expressed as °Brix, varied between 17.7 and 23.50 for all studied cultivars (Table 4).

The equilibrium moisture content (EMC) decreased at higher temperatures and increased with increase in *a_w_*. This finding can be explained by the higher active state of water molecules at higher temperature, which causes the attractive forces between them to decrease (16).

This behavior was also reported in other crops such as dates [28], potatoes [29], apricots and apples [20], and bananas [30]. Generally, their desorption isotherms showed that when the water activity is lower than 0.55, fruit gained relatively low moisture content. However, when water activity values are higher than 0.55, solid solubilization and adsorption promoted a significant increase in product moisture content [31]. At constant water activity, the equilibrium moisture content decreases as the temperature increases. This indicates that the sweet cherry samples are less hygroscopic at high temperature [32–35]. The water activity was generally in the range 0.3-0.4, except for the Burlat (*a*_*w*_ = 0.46) and Napoleon (*a*_*w*_ = 0.44) cultivar fruits which means that the drying will extend their shelf life and can be stored for a long time without any negative effect on their quality.

The hygroscopic equilibrium of sweet cherry fruits was reached in 10 days for desorption and 9 days for adsorption. The hysteresis phenomena occurred at all temperatures and for all five cultivars. Its loop increased with an increase in temperatures [36].

In fact, the predrying of the fruits samples before the adsorption assays may have induced or modified structural changes that result in the deactivation of binding sites previously present [37], therefore lowering the *X_e_* value to a constant *a_w_* in adsorption when compared to desorption (Figure 2).

### 3.2. Optimal Conditions for Storage

Understanding the relationship between moisture content and water activity, a thermodynamic property regarding the interactions between water molecules and the food product matrix, is a key element to predict its stability. Moisture sorption isotherms are important in defining the optimal food dehydration and storage conditions. Additionally, moisture sorption properties impact a wide range of phenomena such as microbial activity, sensory quality deterioration, undesirable enzymatic reactions, food inner structure, and nutrient losses. The optimal relative humidity suitable for storage is calculated and summarized in Table 5. The water activity was generally in the range 0.3-0.4, except for the Burlat (*a*_*w*_ = 0.46) and Napoleon (*a*_*w*_ = 0.44) cultivar fruits. This discrepancy is likely due to their higher sugar content. For this purpose, we determined the optimum equilibrium relative humidity for storage of cherry.

### 3.3. Modeling of the Sorption Isotherms

The experimental data of the five cherry cultivars were fitted to the three previously mentioned models to describe the moisture sorption isotherms. The best fit model is selected based on the minimum values of the mean relative error (MRE) and the maximum of the correlation coefficient (*r*) [38]. The sorption isotherm studies confirmed that the equilibrium data for cherry fruits of all cultivars fit well with the GAB model. The results of nonlinear regression analysis of fitting the sorption equations to the experimental data are shown in Table 6. The results agree with those reported by Moussaoui et al. [39], whose study showed that the GAB equation representing multilayer adsorption describes the sorption and behavior of argan products well. The GAB model also accurately described the adsorption data for three date cultivars [28].

### 3.4. Thermodynamic Properties

#### 3.4.1. Heat of Sorption

**Figure 3:**
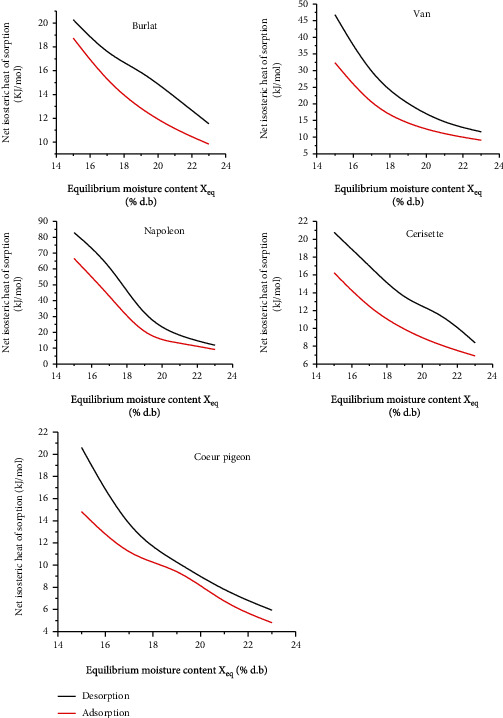
Net isosteric heat of isotherms for five cultivars of cherry.

The evolution of the net isosteric heat of adsorption and desorption of the water content of fruits from the five cherry cultivars are shown in Figure 3. The net isosteric heat of sorption is intensely dependent on moisture content (Figure 3). It decreased rapidly as moisture content increased. The observed increase in net isosteric heat at low moisture content can be attributed to the abundance of active binding sites on the surface of the material. Once they are covered with monolayer water molecules, they become less active, thus generating a lower heat of sorption [29]. When *X*_eq_ is below 24%, *q*_st_ decreased gradually approaching zero, which means that the isosteric heat is equal to the heat of condensation. Furthermore, the corresponding moisture content can be seen as the limit of “bound” water [22]. The dissolution of sugars appears to be the predominant action at higher water content, and the five cultivars show the same features [40].

The net isosteric heat required in the desorption process is greater than that in the adsorption process for all the cherry cultivars. This finding may be due to the higher number of sorption sites on the surface of the materials and the greater bond energy present during the desorption process. Wang and Brennan [41] reported that the desorption heats were significantly higher than the adsorption heats at low moisture contents for many foods. The experiment's results correlated with satisfaction by a 5^th^ order polynomial (Table 7).

#### 3.4.2. Sorption Entropy

**Figure 4:**
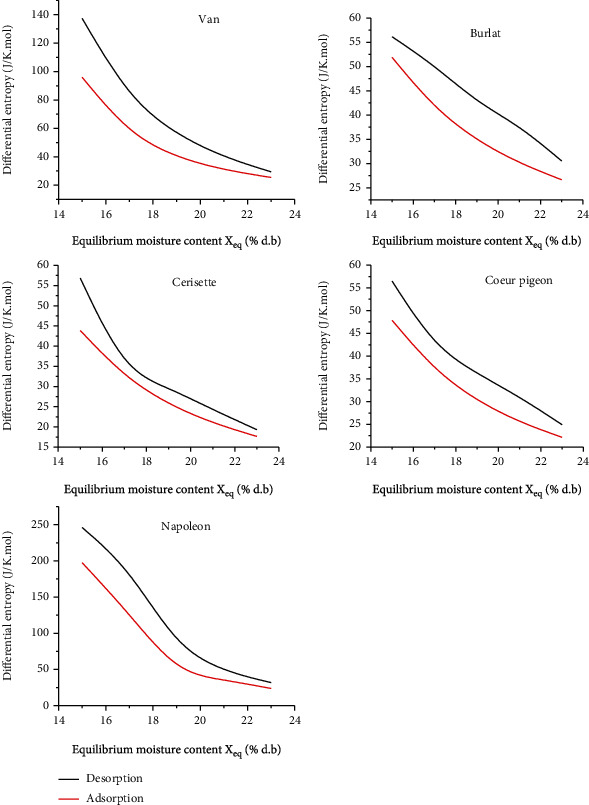
Differential entropy of isotherms for five cultivars of cherry.

The differential entropy of isotherms (Δ*S*) for the five cherry cultivars were derived from the graphical representation of Equation (4). It provides information of the irreversibility of physical phenomena, especially during thermal exchanges, as well as the thermal energy losses (*T* Δ*S*) in the system. Differential enthalpy of sorption of the cherry samples of the five cultivars at the three temperatures is reported in Figure 4. Independently of the cultivar, the differential entropy decreased sharply with a range of 14-24% of the equilibrium water content for both adsorption and desorption. Experimentally generated values are reported in Table 8 with a coefficient *r* equal to the unit and the null value of the mean squared error.

As mentioned, the differential entropy increased strongly with decreasing equilibrium moisture content as the isosteric heat of desorption (Figure 4). Similar findings were reported by Al-Muhtaseb et al. for the starch powders [42], by Madamba et al. for garlic [43], by Goneli et al. for okra seeds and pearl millet grain [44, 45], by Kahyaoglu and Kaya for sesame seeds [46], by Aviara et al. for melon seed and cassava [47], and by Hassini et al. for prickly pear seeds [35].

#### 3.4.3. Enthalpy-Entropy Compensation

**Figure 5:**
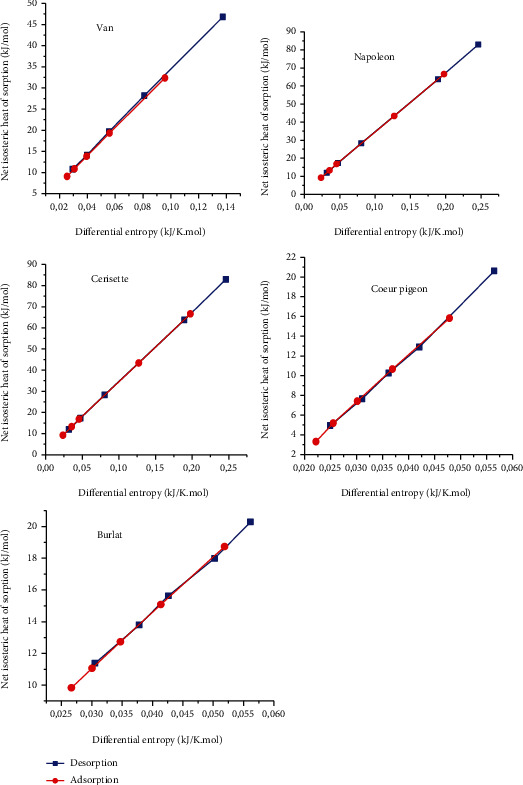
Theory of compensation enthalpy-entropy for the five cultivars of cherry.

Figure 5 shows the compensation theory for the five sweet cherry cultivars. According to this theory, for a specific reaction, enthalpy evolves linearly according to the entropy. All curves generated for the five cultivars aligned with this theory. The isokinetic temperature as well as the free energy, relative to each cherry cultivar, were estimated from the curves (Figure 5) and reported (Table 9). The observed isokinetic temperature values were very close, and they differed from the value of the harmonic temperature and this confirms the enthalpy–entropy compensation theory. This finding validates the criterion of the theory of compensation (Equation (6)). The values of free energy for the five cultivars of sweet cherry were positive indicating a nonspontaneous desorption process. Moreover, isotherms of cherry fruits did not occur spontaneously. The estimated optimum drying temperatures for the five cultivars varied from 50 to 80°C. These temperatures are suitable for drying foods without affecting their quality.

Similar results for enthalpy–entropy compensation were obtained by Beristain et al. for sugar-rich foods (prunes, apricots, figs, raisins, and apricots) [48], Gabas et al. for plum skin and pulp [49], Arslan and Toǧrul for tea [50], Noshad et al. for quince [51], Goneli et al. for pearl millet grain [45], and by Hassini et al. for prickly pear seeds [35].

#### 3.4.4. Spreading Pressure

**Figure 6:**
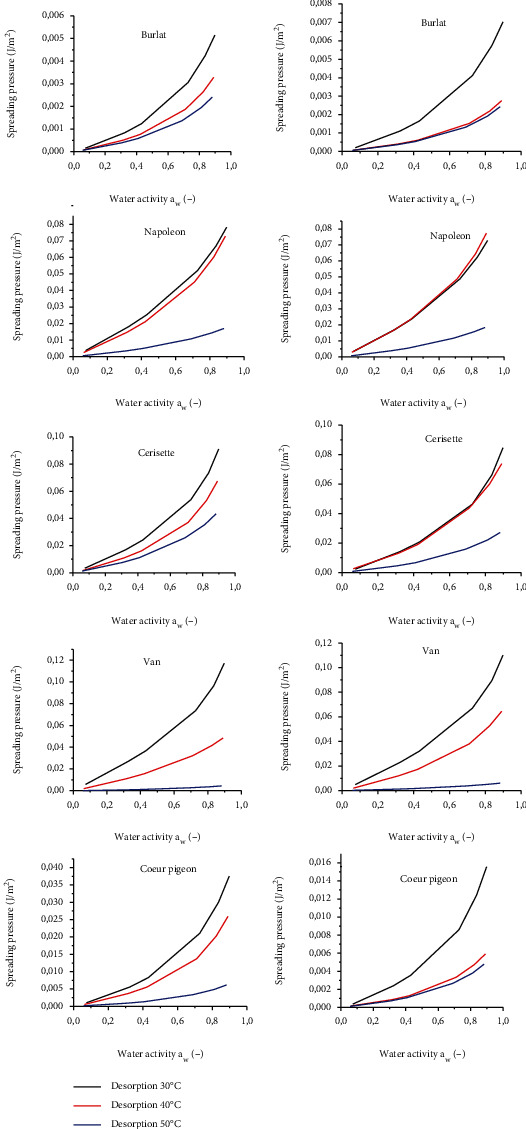
Spreading pressure isotherms at different temperatures of adsorption and desorption for the five cultivars of cherry.

The spreading pressures of samples of each sweet cherry cultivar at different temperatures were determined by the equations (Equations (7) and (8)) and plotted (Figure 6). The spreading pressure values increased as the water activity increased. However, the sinusoidal behavior of the isotherm is doomed to disappear at a given temperature. Therefore, spreading pressure may have a linear dependence versus *a_w_*. The spreading pressure value varied with temperature in all sweet cherry cultivars in both desorption and adsorption processes. The observed high spreading pressure values at lower temperature indicates high affinity for water molecules to active sites, which is most likely due to the structure and the chemical composition of the cherry fruits. The effect of temperature on the spreading pressure values differs depending on the cultivar studied, probably due to the type of structure and the chemical composition fruits from each cherry cultivar. The same behavior was reported by Al-Muhtaseb et al. [42] in starch powders. This assessment allows analogies to be made and differences with other materials to be recognized, giving us a better understanding of the thermodynamic mechanisms of the different cultivars of cherry.

#### 3.4.5. Surface Area

The water surface area of different cultivars of cherry between 30 and 50°C are presented in Table 10. These values were estimated using Equation (9) and the monolayer moisture contents were obtained by GAB model. The results presented in Table 10 indicate that the total surface area available for sorption decreased with increasing temperature. The large surface area of many foodstuffs is due to the existence of an intrinsic microspore structure in these materials [52]. For quinoa grains, Tolaba et al. [53] reported the surface area values of 303.45, 297.85, and 206.5 m^2^ g^−1^ for adsorption, and 349.65, 303.1, and 200.55 m^2^ g^−1^ for desorption, for the temperatures of 30, 40, and 50°C, respectively.

## 4. Conclusion

Efficient processing and storage of sweet cherry powder require reducing its moisture content to an appropriate level by drying. This involves a deep knowledge of physical properties and moisture sorption isotherm.

The equilibrium moisture content was experimentally measured during desorption and adsorption of water in five sweet cherry cultivars at 30, 40, and 50°C. Equilibrium moisture contents decreased as temperature increased at constant water activity. In addition, they also increased with increasing water activity at a constant temperature. Among the models tested to interpret isotherms of sweet cherries, the GAB was the most suitable fit model.

It is well established that most biochemical and microbiological reactions in a given food matrix can be inhibited and the degradation of the food product can be prevented when water activity is < 0.6, and a slight increase in *a_w_* equivalent to an increase of moisture content by 0.1 unit reduces the shelf life of a food product by a factor of 2–3. In this study, the water activity range 0.3-0.4 reported for the five cultivars suggest that the sweet cherry fruits can be stored safely without any biological alteration.

Based, on the isokinetic temperature as well as the free energy, the estimated optimum drying temperatures for the five cultivars were between 50 and 80°C. These temperatures are suitable for drying foods without affecting their quality.

The findings of this research may be important by providing information for understanding the drying behavior and the drying process conditions of sweet cherry fruits from an industrial perspective.

## Figures and Tables

**Table 1:** Standard values of the water activities of the salts used according to temperature [54].

	KOH	MgCl_2_.6H_2_O	K_2_CO_3_	NaNO_3_	KCl	BaCl_2_.2H_2_O
30°C	0.0738	0.3238	0.4317	0.7275	0.8362	0.898
40°C	0.0626	0.3159	0.423	0.71	0.8232	0.891
50°C	0.0572	0.3054	0.4091	0.6904	0.812	0.8823

**Table 2:** Moisture sorption isotherm models used to analyze data for cherry cultivars.

Models	Mathematical expression	Range of validity	References
GAB	$X_{\text{eq}}=\frac{\text{ABC }a_{w}}{\left[1-\text{B}{\text{a}}_{w}\right]\left[1-\text{B}{\text{a}}_{w}-\text{BC}{\text{a}}_{w}\right]}$	Complete	[55]
ENDERBY	$X_{\text{eq}}=\left[\frac{A}{\left(1-\text{B}{\text{a}}_{w}\right)}+\frac{C}{\left(1-\text{D}{\text{a}}_{w}\right)}\right]a_{w}$	Complete	[56]
PELEG	*X*_eq_ = *A*(*a*_*w*_)^*C*^ + *B*(*a*_*w*_)^*D*^	Complete	[57]

**(a):** 

	Sorption isotherms at 30°C					
	*a_w_*	Burlat	Van	Napoleon	Cerisette	Cœur pigeon
Desorption	0.0738	14.47	13.18	14.15	13.01	7.21
	0.3238	17.12	16.83	22.60	14.16	11.18
	0.4317	22.15	32.26	27.96	17.47	16.53
	0.7275	48.23	37.23	42.21	48.21	43.96
	0.8362	78.77	52.33	52.96	53.63	57.49
	0.898	92.88	92.91	91.44	96.63	88.43

Adsorption	0.0738	13.46	11.31	13.70	11.72	7.19
	0.3238	15.48	13.33	20.81	13.57	11.04
	0.4317	19.96	24.78	26.41	16.31	15.66
	0.7275	39.35	33.34	41.41	44.47	42.98
	0.8362	71.78	50.72	48.76	52.54	55.50
	0.898	84.49	85.00	71.93	85.98	81.40

**(b):** 

	Sorption isotherms at 40°C					
	*a_w_*	Burlat	Van	Napoleon	Cerisette	Cœur pigeon
Desorption	0.0626	10.40	12.79	10.36	11.81	8.40
	0.3259	13.08	16.74	19.74	13.30	10.63
	0.4230	16.50	19.40	23.70	16.21	14.29
	0.7100	39.35	27.99	34.97	43.31	43.42
	0.8232	65.93	45.68	49.04	49.25	53.36
	0.8910	76.14	48.15	69.64	74.57	80.49

Adsorption	0.0626	8.14	6.32	8.54	8.14	6.24
	0.3259	10.53	12.38	17.44	12.77	10.13
	0.4230	11.59	15.50	22.85	15.37	13.44
	0.7100	33.22	25.24	31.96	38.68	37.16
	0.8232	60.19	35.24	47.80	46.53	46.18
	0.8910	68.33	42.59	66.48	68.80	67.29

**(c):** 

	Sorption isotherms at 50°C					
	*a_w_*	Burlat	Van	Napoleon	Cerisette	Cœur pigeon
Desorption	0.0572	6.83	11.84	8.66	7.31	8.01
	0.3054	9.61	9.39	18.18	12.90	9.63
	0.4091	15.43	14.51	21.34	15.69	13.58
	0.6904	36.66	25.94	33.57	29.45	41.45
	0.8120	57.29	42.86	41.46	46.91	49.43
	0.8823	60.05	44.28	53.91	70.05	79.72

Adsorption	0.0572	5.23	4.43	7.09	7.80	5.75
	0.3054	7.05	10.64	16.71	11.78	9.99
	0.4091	10.95	13.70	21.19	14.72	16.68
	0.6904	26.56	17.74	32.31	24.26	34.99
	0.8120	52.70	31.25	39.28	43.99	43.73
	0.8823	53.45	40.93	51.21	67.01	65.75

**Table 4:** Characteristics of the fruits used.

Cherry cultivars	°Brix	Moisture %
Burlat	24.00	84.01
Van	24.50	86.11
Napoleon	23.10	77.80
Cerisette	17.70	84.56
Cœur de pigeon	19.80	82.61

**Table 5:** Analysis of the curves of sorption according to a model of 3rd degree.

Cherry cultivars	[*a_w_* (op); *X*_eq_]
Burlat	[0.46; 8.64]
Van	[0.42; 14.38]
Napoleon	[0.44; 20.09]
Cerisette	[0.41; 11.98]
Cœur de pigeon	[0.36; 11.22]

**Table 6:** Estimated parameters of models for cherry cultivars at different temperatures of desorption and adsorption curves.

	Model	Temperature °C	Burlat		Van		Napoleon		Cerisette		Cœur pigeon	
			*r*	RME	*r*	RME	*r*	RME	*r*	RME	*r*	RME
Desorption	GAB	30	0.996	3.829	0.979	7.764	0.992	3.503	0.984	7.591	0.995	4.166
		40	0.993	4.437	0.977	4.274	0.994	3.118	0.987	5.197	0.992	4.834
		50	0.98	6.154	0.977	4.274	0.992	2.758	1	0.942	0.988	5.771
	PELEG	30	0.969	13.167	0.872	22.592	0.997	2.766	0.986	8.78	0.996	4.587
		40	0.997	3.483	0.986	3.936	0.999	0.911	0.986	6.714	0.991	6.171
		50	0.995	3.744	0.989	3.738	0.966	6.782	0.915	11.123	0.971	10.85
	ENDERBY	30	0.995	5.33	0.990	6.479	0.941	11.756	0.99	7.668	0.997	3.669
		40	0.986	7.45	0.984	4.281	0.999	0.675	0.970	9.690	0.987	7.270
		50	0.989	5.522	0.975	5.452	0.968	6.643	1	0.161	0.991	5.974

Adsorption	GAB	30	0.992	4.899	0.992	4.632	0.988	4.305	0.989	5.581	0.995	3.765
		40	0.988	5.401	0.997	1.378	0.993	3.358	0.993	3.688	0.993	3.598
		50	0.972	6.796	0.989	2.673	0.988	3.275	1	1.494	0.992	3.857
	PELEG	30	0.958	13.922	0.994	4.898	0.927	12.716	0.949	14.568	0.994	5.192
		40	0.995	4.3742	1.00	0.638	0.999	1.325	0.991	5.023	0.980	7.599
		50	0.982	6.651	0.941	7.307	0.998	1.390	0.998	0.824	0.996	3.405
	ENDERBY	30	0.991	6.42	0.995	4.647	0.934	12.072	0.988	7.074	0.996	3.958
		40	0.985	7.20	0.999	0.772	0.998	1.818	0.992	4.781	0.990	5.310
		50	0.980	7.045	0.994	2.444	0.998	1.564	1	1.773	0.997	2.954

**Table 7:** Net isosteric heat of isotherms for five cultivars of cherry.

		*q*_st_ (kJ/Mol)	
Desorption	Burlat	−0.0167*X*_eq_^3^ + 1.0982*X*_eq_^2^ − 24.7580*X*_eq_ + 200.7869	*r* = 1
	Van	−0.0531*X*_eq_^3^ + 3.5625*X*_eq_^2^ − 81.969*X*_eq_ + 653.6054	*r* = 1
	Napoleon	0.2284*X*_eq_^3^ − 12.0878*X*_eq_^2^ + 199.4879*X*_eq_ − 959.6137	*r* = 1
	Cerisette	−0.0317*X*_eq_^3^ + 2.0622*X*_eq_^2^ − 45.4246*X*_eq_ + 345.1743	*r* = 1
	Cœur pigeon	−0.0267*X*_eq_^3^ + 1.8053*X*_eq_^2^ − 41.1935*X*_eq_ + 322.137	*r* = 1

Adsorption	Burlat	−0.00893 *X*_eq_^3^ + 0.6078 *X*_eq_^2^ − 14.3947 *X*_eq_ + 128.0349	*r* = 1
	Van	−0.0668 *X*_eq_^3^ + 4.2554 *X*_eq_^2^ − 91.1603 *X*_eq_ + 667.8193	*r* = 1
	Napoleon	0.0288 *X*_eq_^3^ − 0.5466 *X*_eq_^2^ − 18.064*X*_eq_ + 364.0534	*r* = 1
	Cerisette	−0.0114 *X*_eq_^3^ + 0.7615 *X*_eq_^2^ − 17.622*X*_eq_ + 147.5421	*r* = 1
	Cœur pigeon	−0.0173*X*_eq_^3^ + 1.073*X*_eq_^2^ − 22.8434*X*_eq_ + 174.2007	*r* = 1

**Table 8:** Differential entropy of isotherms for five cultivars of cherry.

		Δ*S* (J.Mol^−1^.K^−1^)	
Desorption	Burlat	−0.0496X_eq_^3^ + 3.2554X_eq_^2^ − 72.9183X_eq_ + 582.7241	*r* = 1
	Van	−0.1619*X*_eq_^3^ + 10.8656*X*_eq_^2^ − 249.7396*X*_eq_ + 1984.3723	*r* = 1
	Napoleon	0.7204*X*_eq_^3^ − 38.241*X*_eq_^2^ + 634.6694*X*_eq_ − 3098.227	*r* = 1
	Cerisette	−0.0969*X*_eq_^3^ + 6.285*X*_eq_^2^ − 137.9286*X*_eq_ + 1038.5512	*r* = 1
	Cœur pigeon	−0.0993*X*_eq_^3^ + 5.9688*X*_eq_^2^ − 121.6653*X*_eq_ + 873.4211	*r* = 1

Adsorption	Burlat	−0.0267*X*_eq_^3^ + 1.8093*X*_eq_^2^ − 42.6014*X*_eq_ + 373.766	*r* = 1
	Van	−0.208*X*_eq_^3^ + 13.2231*X*_eq_^2^ − 282.832*X*_eq_ + 2064.5612	*r* = 1
	Napoleon	0.095*X*_eq_^3^ − 2.03*X*_eq_^2^ − 48.69*X*_eq_ + 1067.3451	*r* = 1
	Cerisette	−0.0336*X*_eq_^3^ + 2.2502*X*_eq_^2^ − 51.792*X*_eq_ + 427.987	*r* = 1
	Cœur pigeon	−0.0302*X*_eq_^3^ + 2.0297*X*_eq_^2^ − 47.1964*X*_eq_ + 400.8916	*r* = 1

**Table 9:** Values of isokinetic temperature and free energy for five cultivars of cherry.

		*T*_*β*_(K)	Δ*G*_*β*_ (J.Mol^−1^)
Desorption	Burlat	362.21	725.04
	Van	332.00	1231.44
	Napoleon	329.51	1606.37
	Cerisette	344.6043	1243.93
	Cœur pigeon	349.532	1321.53

Adsorption	Burlat	352.8082	455.20
	Van	330.30	731.003
	Napoleon	329.062	1594.90
	Cerisette	355.72	665.5821
	Cœur pigeon	351.64	1021.0758

**Table 10:** Water surface area (*S*0) of cherry fruit at different temperatures.

	Desorption			Adsorption		
T(°C)	30	40	50	30	40	50
Burlat	174.65	107.8	84.35	161	84.35	75.95
Van	179.2	153.65	133.7	151.9	107.1	101.5
Napoleon	179.1	108.92	78.75	178.75	101.15	73.85
Cerisette	213.92	165.60	122.15	204.4	95.9	79.8
Cœur pigeon	214.03	151.2	78.05	176.4	81.2	49.00

## Data Availability

The data used to support the findings of this study are available upon request from the corresponding author.
